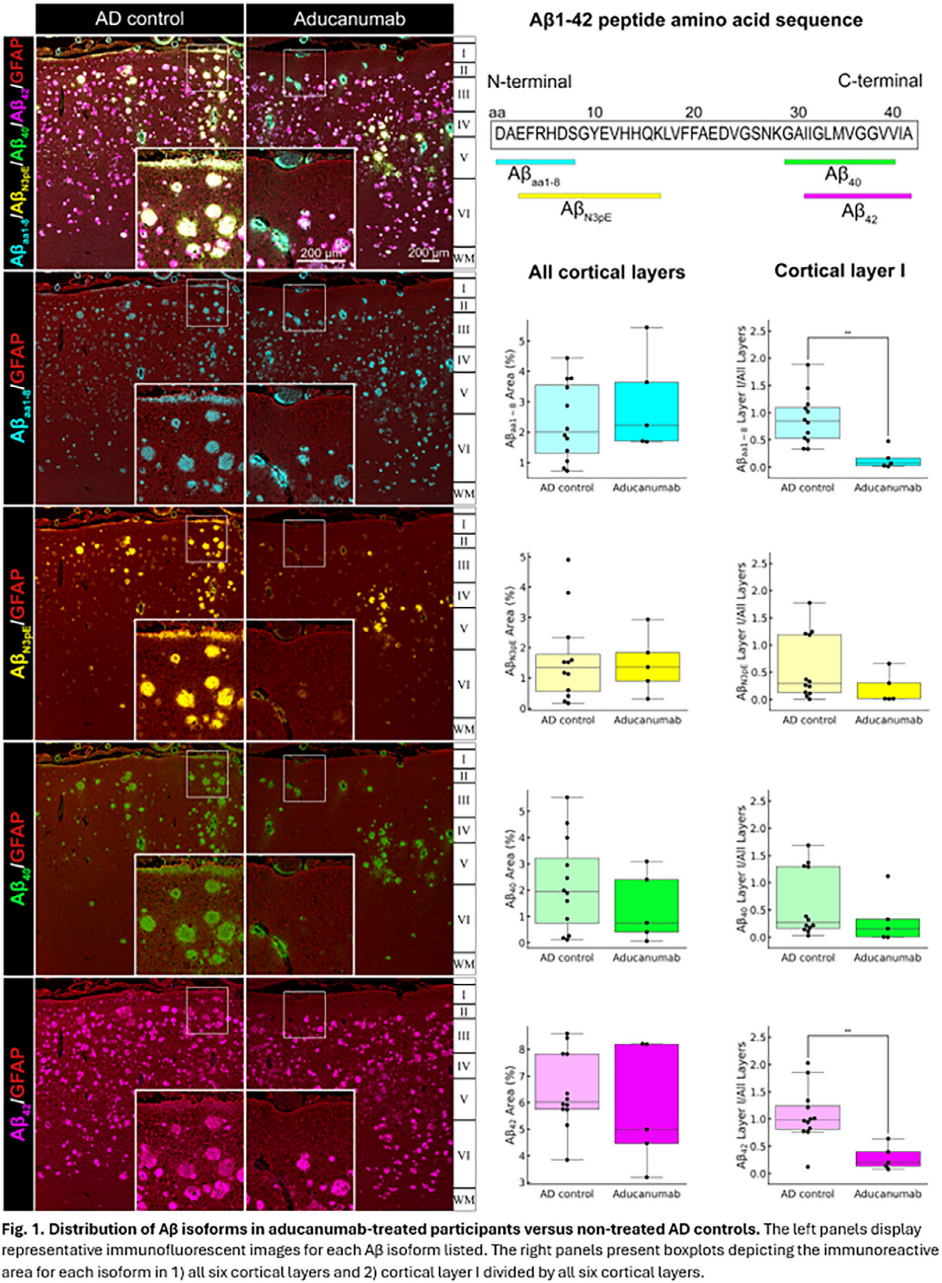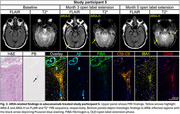# Superficial Cortical Aβ Clearance and ARIA Pathology in Aducanumab‐Treated Alzheimer's Disease

**DOI:** 10.1002/alz70855_105890

**Published:** 2025-12-24

**Authors:** Baayla D.C. Boon, Yoav D Piura, Christina M. Moloney, Jessica L Chalk, Sarah J. Lincoln, Matthew H. Rutledge, Derek R. Johnson, Dennis W. Dickson, Aivi T. Nguyen, R. Ross Reichard, Jonathan Graff‐Radford, David S. Knopman, Neill R. Graff‐Radford, Melissa E. Murray

**Affiliations:** ^1^ Mayo Clinic, Jacksonville, FL, USA; ^2^ Amsterdam UMC, location VUmc, Amsterdam, Netherlands; ^3^ Mayo Clinic in Florida, Jacksonville, FL, USA; ^4^ Mayo Clinic, Rochester, MN, USA; ^5^ Department of Neurology, Mayo Clinic, Rochester, MN, USA; ^6^ Department of Laboratory Medicine and Pathology, Mayo Clinic, Rochester, MN, USA

## Abstract

**Background:**

Monoclonal antibodies targeting amyloid‐β (Aβ), including aducanumab, have been tested to treat Alzheimer's disease (AD). However, limited data exist on neuropathology following treatment and the effects of amyloid‐related imaging abnormalities (ARIA). We report on such data from five aducanumab‐treated study participants with AD of whom two experienced ARIA.

**Methods:**

Aducanumab‐treated study participants who came to autopsy (*n* = 5) were matched to AD controls (*n* = 12) based on the presence/type of autosomal dominant AD mutation, *APOE* genotype, age at cognitive symptom onset, and sex. We assessed cognitive measures, ^18^F‐florbetapir PET centiloid, ARIA risk factors, and AD neuropathologic change. Using multiplex immunofluorescence, brain regions affected along Thal Aβ phases were stained for Aβ isoforms and phosphorylated tau, as well as for ARIA‐associated markers, including Perls’ Prussian blue to detect ferric iron, fibrinogen‐α for blood brain barrier integrity, membrane attack complex (C5b‐C9) for complement activation, and activated microglia (IBA1, CD68).

**Results:**

Study participants included four males and one female, all carrying at least one *APOE ε4* allele. Two participants carried a *PSEN1 NM_000021.4:c.817G>A*, p.Glu273Lys mutation. Cumulative aducanumab dosage ranged between 5–241 mg/kg, with death occurring 5–41 months after the last infusion. Amyloid burden on PET declined in all participants (range 15‐100 centiloids). Two participants experienced ARIA. Compared to AD controls, Aβ_aa1‐8_ and Aβ_42_ were selectively reduced in cortical layer I of aducanumab‐treated study participants (*p* <0.05) but not in the total cortex, as shown for the middle frontal cortex in Figure 1. No differences were observed in phosphorylated tau burden. Prussian blue‐stained hemosiderin was present in both treated and untreated AD cases. However, in participants with ARIA, hemosiderin accumulated in superficial cortical layers near CAA‐laden meningeal and penetrating vessels, which also exhibited extensive complement and microglial activation (Figure 2).

**Conclusions:**

Aβ clearance and ARIA‐related findings were localized predominantly in superficial cortical layers, suggesting that aducanumab biodistribution is more pronounced in these regions rather than deeper cortical layers.